# Determinants, Discriminants, Conserved Residues - A Heuristic Approach to Detection of Functional Divergence in Protein Families

**DOI:** 10.1371/journal.pone.0024382

**Published:** 2011-09-12

**Authors:** Kavitha Bharatham, Zong Hong Zhang, Ivana Mihalek

**Affiliations:** 1 Bioinformatics Institute, Agency for Science, Technology and Research, Singapore, Singapore; 2 School of Biological Sciences, Nanyang Technological University, Singapore, Singapore; University of South Florida College of Medicine, United States of America

## Abstract

In this work, belonging to the field of comparative analysis of protein sequences, we focus on detection of functional specialization on the residue level. As the input, we take a set of sequences divided into groups of orthologues, each group known to be responsible for a different function.

This provides two independent pieces of information: within group conservation and overlap in amino acid type across groups. We build our discussion around the set of scoring functions that keep the two separated and the source of the signal easy to trace back to its source.

We propose a heuristic description of functional divergence that includes residue type exchangeability, both in the conservation and in the overlap measure, and does not make any assumptions on the rate of evolution in the groups other than the one under consideration. Residue types acceptable at a certain position within an orthologous group are described as a distribution which evolves in time, starting from a single ancestral type, and is subject to constraints that can be inferred only indirectly. To estimate the strength of the constraints, we compare the observed degrees of conservation and overlap with those expected in the hypothetical case of a freely evolving distribution.

Our description matches the experiment well, but we also conclude that any attempt to capture the evolutionary behavior of specificity determining residues in terms of a scalar function will be tentative, because no single model can cover the variety of evolutionary behavior such residues exhibit. Especially, models expecting the same type of evolutionary behavior across functionally divergent groups tend to miss a portion of information otherwise retrievable by the conservation and overlap measures they use.

## Introduction

In the standard approach to computational analysis of proteins, the first step is detection of their functional parts through comparative analysis of homologous sequences. As databases fill with protein sequences from well beyond a handful of model organisms of a single genotype, this preliminary step is becoming increasingly rewarding both in terms of feasibility and of reasonably high resolution for most proteins of technological interest.

Two types of evolutionary behavior are typically sought in a comparative analysis of a protein family: conservation across several groups of homologues, and specialization within each group. The former is of interest for understanding structural and folding features of the class of proteins as a whole, while the latter becomes interesting in an attempt to control a particular set of paralogues, such as in designing a highly specific drug. The latter is also the topic of this work. We discuss a class of heuristic methods designed to detect functional specialization without reconstructing the underlying sequence of evolutionary events.

If gene duplication did not exist, we could only observe variability across orthologues from different organisms. The discussion thus naturally starts with the methods to score residue conservation [1]. Historically they arrived first, ranging from simple majority fraction [2] to information entropy [3]–[5] and entropy related methods [6], to full-blown statistical estimation of the mutability of residues leading to the observed set of sequences [7], [8]. Such methods work well in detecting the folding core of a protein [9], the catalytic site of an enzyme, and somewhat less reliably, the protein-protein interfaces shared by all homologues [10], [11]. Their performance is affected more strongly by the pre-processing stage (in which an informative set of wild-type, mutually orthologous, sequences must be selected), then by the choice of method itself [12].

The specialization of duplicated genes is the necessary condition for their parallel existence, and the methods to detect it on the protein level followed shortly [13]–[16]. Several major ways of treating this problem have been put forth, differing mainly in (i) the way they handle the classification of proteins into orthologous groups, and (ii) the underlying model of evolution they incorporate. The first issue has been dealt with by taking the classification as an input [17], by using the similarity tree as the classification generator [13], [14], [18], or by adopting a midway solution in which the tree is provided by the application, but the relevant division into subtrees is decided on by the user [19].

In this work, we would like to put some emphasis on the way an evolutionary model is built into a specificity scoring function. As an example, a popularly quoted evolutionary trace method, ET [14], in its original formulation assumes that a functionally important position will be completely conserved in each of the compared groups of sequences, albeit as a different amino acid type. If the groups in question are paralogous, this becomes a very strict model of evolution, in which even after the duplication and specialization event(s), each gene maintains the same degree of evolutionary pressure at each site. (For a recent remedy see [20]). This model appears in the literature in several forms (“conservatism-of-conservatism” [16], “constant but different” [21], “type II functional divergence”[22], as evenly weighted correction to entropy from each branch in the tree [6], as a log-likelihood of a type conditioned on tree [18], Venn diagrams [23]). Conversely, mutual information (MI [17], [24], another very successful import from information theory) requires that each group of orthologues adopts a set of evolutionary constraints that are systematically different from those of all other groups, irrespective of the degree of conservation within each group. However, it mirrors “conservatism-of-conservatism” in conditioning the expected behavior in one group, on the behavior in another.

Recently, ever more voices appear in the literature, pointing out that the evolutionary behavior in paralogous groups may be completely unrelated. Variously termed “type I functional divergence”[22] or “heterotachy”[25], this type of behavior has been discussed in genetics literature for at least a decade [15], and used increasingly in detection of family specific positions on a nucleotide or peptide sequence [22], [26]–[29].

Finding the “type I - type II” terminology somewhat lacking in descriptive power, we use the term “determinants” for the positions that are conserved in one group, but evolve at various rates across paralogues (since they determine the function of the group in which they are found conserved), and “discriminants” for the positions that vary at comparable low rates across all groups (because they work as a unique tag for each of the groups). A determinant position, then, is a property of a single group, while a discriminant is a property of the family as a whole.

The central claim of the work is that there is no “magic bullet” combination of conservation and overlap scoring functions that can solve the problem of detection of functional specialization. Rather than comparing various proprietary combinations thereof, we suggest looking at their ingredients, one at a time, with everything else fixed, and considering how well they describe documented cases of functional divergence. We also stress the fact that scoring functions, wittingly or not, often encompass an evolutionary model (an assumption of discriminant behavior) that cannot be applied across the board. While discriminants can be commonly found in catalytic sites of enzymes, they are more of an exception than a rule in a general case of functional divergence.

When dealing with real-life data there are many additional practical problems that need to be resolved, and diverse sources of information that need to be collated. The estimation of the reliability of the alignment in the neighborhood of the residue of interest (perhaps through the conservation in the neighborhood window [30]), treatment of gaps, unsupervised detection of orthologous groups [31]–[34] mapping onto the structure [35]–[38], as well as detecting synergistic co-evolutionary events [39], [40] are all important issues, but downstream or complementary to the basic specialization scoring framework we propose to discuss here.

In the following section (Method), we lay out the framework for discussion of overlap and conservation measures. Therein we also outline the incorporation of residue exchangeability in the description, and show how these basic ingredients combine into various specialization scoring functions. In the Results section we take a look at several examples of specialization among families of paralogous proteins, and discuss where the responsible residues fall on the conservation/overlap grid. We consider the options available in building a scoring function at a heuristic, phylogeny independent level, and propose a strategy that allows us to move on from catalytic sites of enzymes to more general cases of protein functional divergence.

## Methods

Let us first consider the case of the comparison of a family consisting of two paralogous groups of proteins only. The generalization to the case of a multimember family will be straightforward. We consider one position in the alignment of protein sequences at a time, and assume that each group is represented by a fair sample of orthologous proteins from a comparable set of species. All the scores we discuss are relative - they are meaningful only in the context of a given multiple sequence alignment. Their absolute values have no intrinsic meaning.

We center the discussion around two independent types of information: within-group conservation, and overlap in the choice of residue type across the two groups. Various methods proposed in the literature to score functional specialization differ mostly in how they extract this information, and which combination thereof they take as the key property to be detected.

**Figure 1:**
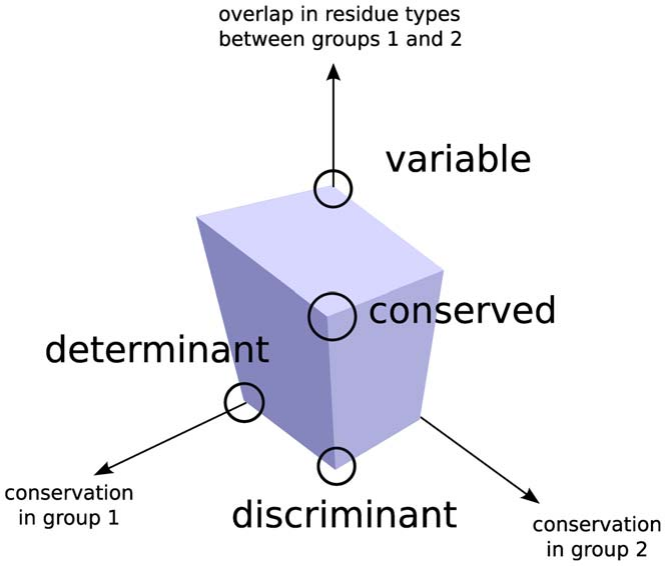
The main components of the information available from comparative analysis of two groups of paralogous sequences. The nomenclature we use in this paper for the three main types of behavior is also indicated.

To be more specific, we refer to Fig. 1. Assuming that we have devised a way to score the conservation and overlap in the choice of residue types, and that the assigned score lies in a finite interval of values, say between 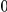 and 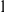, we can then assign to each alignment column a triplet of values (conservation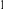, conservation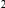, overlap). Their extremal combinations then correspond to the corners of the cube of side 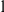. Thus the triplet 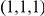 corresponds to the column which is conserved and consists of the same residue type in both groups, 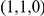 to the column which is conserved in each group but different between the two (discriminant), 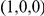 to a position which is determinant of the group 1, and so on. Notably, in this way of representing the information, the completely variable position gets assigned the triplet 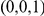, which is diametrically opposite to the triplet representing a fully discriminant position (not a fully conserved one).

What various scoring schemes do is score the positions according to their proximity or distance from one of the corners. We will return to the question of incorporating these three numbers into a single score after discussing ways of quantifying conservation and overlap.

### The model

We assume that we have two samples of sequences from two functionally distinct groups of orthologs, 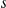 and 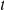. The two samples are fair and cover the same evolutionary breadth in both groups. The two groups can be unambiguously aligned, so it makes sense to speak of position 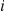 in the context of both groups. To each position 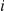 we assign the probability of being occupied by an (amino acid) type 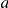, which belongs to the standard 20-letter alphabet. The probability, which is different for the two groups, is estimated by its frequency, 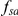 (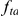 in the other group). It should be kept in mind that these numbers are, in general, different for each position 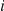, but we will suppress the index, not to burden the notation.

The model also takes that in the absence of any structural or functional constraints, distribution of residue types acceptable at position 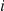, 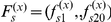, evolves from time 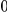 to time 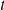 according to the transition probability matrix 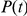


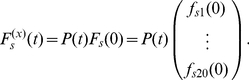
(1)


We are using the superscript 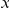 to indicate that this is the frequency distribution expected in the average case of a freely evolving position. We assume here that for each position the amino acid type from the last common ancestor can be determined, so 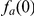 is non-zero for a single type 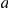 only. The element of this matrix, 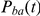 is the probability of the amino acid type indexed by 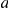 mutating to the one indexed by 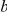 in time 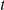. The matrix 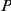, in turn, is generated by the rate matrix 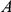 [41],
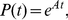
(2)with 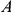 time independent. This comes handy, because it enables us to evaluate 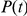 for an arbitrary point in time. Various estimates for the matrix 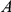 that reproduces the average mutational propensity of residues observed in nature can be found evaluated in literature. The replacement matrix used here was derived by Veerassamy *et al.* [42], by fitting onto the BLOSUM series of matrices [43]. (For alternative methods to derive a rate matrix see for example [44] and references therein.)

For very long times 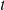, any initial distribution ends up transformed into a stationary distribution 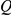,
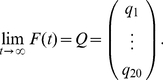
(3)


Distribution 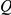 is fixed by the choice of matrix 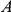. This distribution is the background distribution in the model - the distribution that any initial distribution would eventually turn into, if free of all constraints.

### Within-group conservation

Among the measures typically used to estimate the variability of residue types [1] the information entropy proves to be particularly robust. In the class of the conservation scoring functions that ignore exchangeability of residues, it has no serious competitor, and it is the method we choose to use here as a model which ignores similarity of amino acid types:
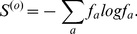
(4)


The sum in this expression runs over the standard alphabet of 20 amino acid types 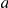, and the superscript 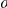 refers to the observed value. (Note that we will be contrasting the expected values, 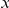, as in Eq. 1 with the observed ones, 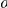, as in the equation above. The expressions without either superscript refer to both.) Various authors prefer different bases for the logarithm, but the choice makes no qualitative difference. To keep the values in the 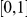 interval, one may use the alphabet size as the base. In the implementation discussed below, we rescale 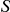 so that 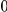 corresponds to the minimum entropy observed within a group, and 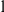 to the maximum. Technically, this number measures the variability of a position. If rescaled to 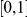, it is a matter of taking a complement, 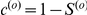 to obtain a number which is 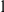 for completely conserved positions, and 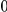 for maximally variable ones.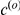, then, measures conservation.

#### Including exchangeability of residue types

The problem with 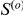 as a measure of variability is that we semi-intuitively expect that some mutations (such as acidic residue to a non-polar one) indicate more variability than the others (such as mutation of one type of acidic residue to the other). The expression in Eq. 4 is blind to that distinction. In literature, several expressions for measuring residue conservation in a model with exchangeable amino acid types have been put forth [1], most based on comparison with the equilibrium distribution of amino acid types, 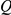, Eq. 3, or some way of incorporating pairwise similarity matrix, such as BLOSUM [1], [30], into the scoring scheme.

As prototypical of these appears Kullback-Leibler divergence
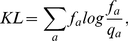
(5)a measure of difference between two distributions, 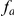 and 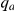 in this case. 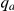 from Eq. 3 is sometimes replaced by an average distribution in the alignment.

Jansen-Shannon divergence, a symmetrized and smoothed version of Kullback-Leibler, has been successfully used by Capra and Singh [30], [45]

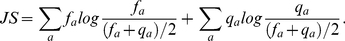
(6)


A potential problem with these types of scoring, as noted by de Vries *et al.* [46] (and again in [47]), is that it drives the correction in a counterintuitive direction: as an example, when completely conserved, a relatively rare residue like tryptophan will end up with a higher score (that is, estimated under higher evolutionary pressure) than isoleucine under the same circumstances. Which should be surprising - given isoleucine's high propensity to mutate to valine or leucine, the absence of “easy” variability should indicate a higher pressure than in the case of tryptophan.

Ultimately, the question is what is it that we are trying to measure - the distance from the (very distant) stationary distribution, or the relative strength of constraints on mutation on one position with respect to another? A direct measure for the latter might be difficult to construct. Instead, we note that one trait that the positions in the alignment have in common is the time they took to diverge from their common ancestral sequence. As an estimate of that time we take the effective time 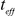, described below, Eq.15. To include into conservation score our knowledge that some residues are more likely to mutate than others, we propose modifying the entropy score, calculated directly from the observed frequency distribution, by its expected value for the freely evolving case:
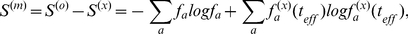
(7)where 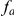 stands for the frequency observed in the alignment, and 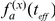 for the expected frequency of the type 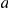 in time 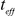, had it been evolving freely from a single ancestral type.

### Overlap of residue type distributions belonging to two protein groups

When comparing two paralogous groups of proteins, labeled 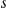 and 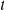, any expression that results in 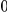 for two distributions with no common elements, and continuously changes to 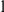 as the two become increasingly similar, is a valid measure of their overlap. (The opposite assignment, 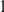 for non-overlapping, 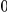 for identical distributions, is equivalent, because it can always be negated and shifted by one to recover the scoring on the 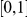 interval.) Similarly, if the upper score is different from 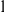, it can always be rescaled, provided that the upper value is a constant, independent of the distributions under consideration.

In this work we suggest using
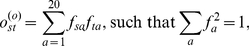
(8)where index 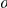 again stands for the observed value, and 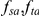 are the frequencies of residue type 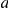 in protein groups 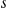 and 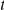 respectively.

Other possibilities include
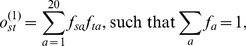
(9)sum of squared differences (*GroupSim* in the original publication [30])
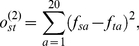
(10)


Kullback-Leibler divergence between the distributions seen in two groups (“relative entropy between groups” in [31]),
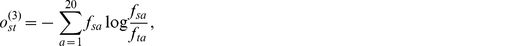
(11)or its symmetrized, Jensen-Shannon, cousin (“sequence harmony” in [48]),
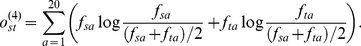
(12)


Some of the overlap measures do better job in separating the two features - conservation and overlap of distributions. Thus 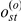 falls naturally between the values of 0 and 1, and is equal to 1 for identical distributions irrespective of their variability. On the contrary, 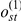, Eq. 9, assigns 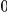 to the overlap of two distributions without any common elements, as expected, but the value assigned to identical distributions depends on their spread. Similarly, 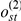, while universally equal to 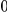 for identical distributions, assigns to two distributions without a common element a number that is dependent on their variability. Though there is no reason to assume that any of these measures is inappropriate for its task, we will adhere to 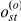 as a measure which separates the conservation and overlap, as it enables to trace the source of information coming from an alignment. 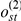 will be used as a representative of measures which do not strictly separate the two.

#### Mutual information

As a special case of a method measuring the overlap in the residue type choice (or, rather, the absence thereof) we highlight mutual information (MI) between the amino acid type and division into groups. The measure is conceptually different from the rest, because it does not compare any two within-group distributions, but, rather, measures how precisely residue types assort themselves into bins provided by the functional groups:
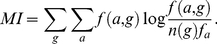
(13)


Here 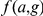 stands for the frequency of 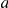 appearing in group 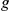, relative to the frequency of all other observed assignments, and 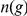 is the relative size of the group 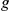, in terms of the number of sequences, compared to the size of all groups combined. Among other interpretations, it can be viewed as Kullback-Leibler divergence, this time measuring the difference of the observed joint probability 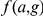, from the value it would have if 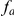 and 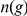, that is, type and grouping into orthologous groups, were independent. This score rewards regular assortment into families other than the one under consideration, which makes it the ultimate discriminant model-incorporating measure. The method is well backed up by the underlying statistical theory, and does its job exactly as it was designed to do, and we use it here to illustrate further that the problem lies with the model of evolution it incorporates, rather than with the overlap measuring function itself.

We also note that mutual information *can* be used as two-distribution overlap, in a way very similar to the rest of the overlap measures described above, if we make the sum over groups 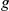 in Eq. 13 run over 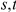 only. This way of using MI is further explored in Text S1, with the conclusion that it does not bring in any universal advantage over other overlap scoring functions.

#### Including exchangeability of residue types

In a way analogous to the modification of entropy, 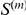, for the case of estimating conservation, Eq. 7, we suggest modifying the overlap measure to incorporate the exchangeability of residue types:
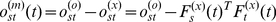
(14)where 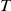 indicates transpose, 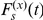 is evaluated according to Eq. 1, and 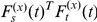 is thus the size of the overlap we would expect in a freely evolving case. As in the case of 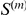, this type correction could in principle be applied to any of conservation and overlap measures in this basic “observed minus expected” form.

### Estimating the effective time since the last common ancestor

As an estimate of the “current” time (the time from the last common ancestor), we take the average time each position would take to evolve freely, and reach the maximal overlap with the observed distribution:
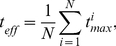
(15)where 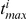 maximizes the overlap between 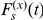 and the observed distribution at the position 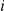. The majority type at position 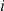 is taken as the ancestral type. In the case of a tie (two types being equally represented and in larger fraction than the rest of the types) we choose as the ancestral the type that produces the larger overlap with the observed distribution.

### Construction of a specialization scoring function 1: Adding conservation and overlap measures

When forced to assign a single number to the functional specificity of a residue, the methods proposed in literature can be viewed as choosing the point of origin on the cube in Fig. 1 from which they score the positions in an alignment, and then rank the residues by either the distance or the proximity to this point of origin. Thus a conservation algorithm scores the residues by the distance from the 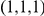 point (the smallest distance indicating the highest conservation)
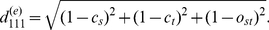
(16)


Indices 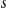 and 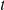 refer to the two groups under consideration. The superscript 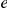 is used to distinguish this, Euclidean, distance, from the linear combination we introduce below. The conservation 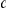 is the complement of variability measured by the information entropy 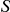, 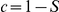. We use the two interchangeably. (In particular, we find the conservation handy for visualization purposes, as in Fig. 1.) A typical discriminant seeking algorithm is looking for points as close to 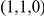 corner as possible [24]

**Figure 2:**
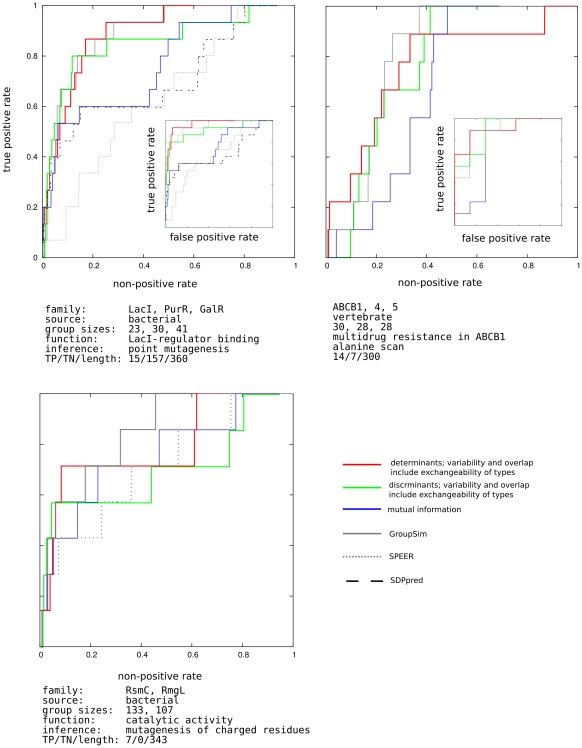
ROC curves for small molecule binding cases. y-axis: true positive rate - fraction of experimentally determined specific resides above threshold. x-axis: non-positive rate - fraction of residues not tested in the experiment. The residues are ordered according to a specificity scoring method. Moving the threshold down the list determines the values plotted int the graph. Inset: x-axis: true positive rate - fraction of experimentally determined specific resides above threshold. x-axis: false positive rate - fraction of residues determined experimentally to be non-specific. The methods tested are indicated in the figure legend. For each family, panel caption lists the families considered (contrasted) in the analysis, taxonomical breadth of source organisms, number of sequences in each group, function tested in the experiment, as well as the method of its inference. The resulting number of true positives (specificity determinants), true negatives, and the length of the target sequence are also listed.



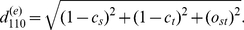
(17)Using a distance from the 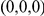 corner (that is the deviation from perfect non-overlap of two non-conserved columns corresponding to the same position in two families) as a measure of specialization also seems appealing (see Results, subsection “Specificity determinants of interferon receptor 2”).

The decision we have to make here is whether to take this, Euclidean, way of adding contributions literally (as suggested, for example, in [49]) or perhaps use a linear combination [45]:
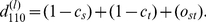
(18)


### Construction of a specialization scoring function 2: Building in a model of evolution

One point that we would like to emphasize here is that once we write an expression such as Eq. 17, we have already committed to the model of functionally discriminant residues - the residues that are conserved in all groups will fare better than the ones that are conserved only in the target group of paralogues.

If, however, we do not expect the specificity determining residues to be conserved in other groups, besides our target group (as is often the case, see Results section below), we should not enforce it in the score either.

Thus, we consider two models of evolutionary behavior of residues, and their incorporation in the overall conservation score - functional discriminants
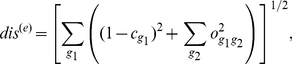
(19)and functional determinants
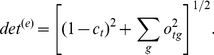
(20)


The sums in the above two equations run over all groups 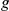 of paralogous proteins present in the analysis. The target group is labeled by 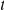. In both cases the smaller score indicates greater specificity. Note that in the case of determinant scoring function, Eq. 20, the requirement on conservation is imposed only in the target group, as is the requirement on overlap between the target group and the remaining groups - the overlap between the pairs not involving 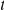 is immaterial.

As noted above, using the Euclidean distance is not the necessary choice. In the following we will also consider linear combinations:
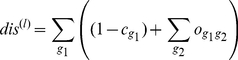
(21)for functional discriminants, and
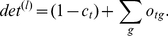
(22)and for functional determinants.

## Results

Our choice of the test set is guided by the following limiting criteria: (i) The functional divergence has experimental backup, through a systematic and unbiased study at the residue level, preferably providing both positive and negative results, related to a well defined phenotype. (ii) The paralogues in question are similar enough so the alignment itself is not an issue. (iii) For all groups in question a reasonably large and diverse number of sequences can be found, from a taxonomically comparable set of species. And last but hardly the least, (iv) we would like to discuss cases more general than the specialization of catalytic pockets of enzymes, such as specialization of protein-protein interaction sites.

In the following sections we divide the examples available in the literature into two groups, roughly corresponding to the cases of divergence in the binding sites of small ligands, and a functional shift involving protein-protein interaction (or its loss).

In all cases we consider the ability of different methods to “detect” - that is, to score highly - residues known to be involved in the specific function of a group by contrasting one or more paralogous groups of proteins.

To keep the discussion compact, for the detailed description of each system we refer the reader to the original publication we derive our test set from.

### Small ligand binding

First, we compare the performance of different specialization scoring schemes for cases where the difference between groups stems from the change in the nature of a small ligand binding site. This is the type of scenario where we are the most likely to encounter the “discriminant” types of positions: binding of a small ligand does not allow much freedom in the residue type choice. Different ligands, however, require different residue types. In such cases mutual information is expected to be a good measure for their detection.

In one of the most thorough point-mutational studies of a protein we have up to date, Suckow and collaborators [50] mutated almost all positions in E. coli lactose inhibitor (LacI) from its wild type to 12 alternative amino acid types, and divided the resulting phenotypes into five distinct groups [51]. The phenotype we are particularly interested in is the loss of inducer response - the trait that distinguishes LacI from its paralogous relatives, purine and galactose repressors (PurR and GalR). The size of this systematic study provided a precious set of true negatives, shown in the inset of the first panel, Fig. 2. In the main panel, the standardly used ROC curve, using residues not explicitly known to be involved in the specific function as the set of “negatives.” The behavior of different scoring methods indicates that while several of the specific residues behave as discriminants, the rest do not, and mutual information fails to locate them. Accordingly, the discriminant scoring function, shown in green, starts detecting specific residues sooner than the determinant one (red), but is, after certain threshold depth, taken over.

As our next test case we take an ABC transporter responsible for development of multidrug resistance was analyzed through a mutational scan of transmembrane domain 11 of mouse orrthologue, by Hannah *et al.* [52]. The related groups of orthologues used are ABCB4 and ABCB5. Compared to the LacI case, the size of the study was small. Both TP and TN sets might be incomplete here. However comparing the ability of different functions to pick up the confirmed true positives from confirmed true negatives shows the ability of determinant model to enrich the top scoring portion of the residues with confirmed TP cases.

The E. coli methyltransferase RsmC was studied by Sunita *et al.* [53]. Charged residues, demonstrated therein through alanine mutagenesis to be involved in catalysis, are used as the true positive set. The paralogous family consists of bacterial RlmG proteins, with different substrate specificity. The nonspecific residues were not explicitly tested in the study.

The sequences used in the alignments, as well as the set of functional residues (as well as negative controls, when available) can be found in Materials S1. Residues conserved across all groups were never considered to be a part of “positive” set of specificity conferring residues.

In all cases the performance of related earlier methods GroupSim [30], SPEER [54], and SDP [55] is shown on the same graph. (Absence in the graph indicates cases when a method does not provide a prediction). These methods have on their own been successfully compared with other, earlier approaches. GroupSim, uses Jensen-Shannon divergence, Eq. 6, as the conservation, and squared difference, Eq. 10, as an overlap measure, combined linearly into a single score (see Methods). The two quantities are not scaled to 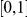 interval as we do here, and additional conservation filter is imposed on the neighboring residues. SDPpred is an elaboration on the mutual information approach, Eq. 13, that additionally estimates the statistical significance of the assigned score. The exchangeability of the residue types is incorporated into the significance calculation. SPEER uses rate4site [7], a phylogeny based method that on its own uses exchangeability in estimating prior mutational probabilities, to estimate difference in evolutionary rates among groups, and linearly combines it with Euclidean distances based on amino acids' physico-chemical properties, and Kullback-Leibler, Eq. 5, type of conservation score. All implementations were used with their default choice of parameters. The problem that is encountered in discussion of these methods is their compounding of conservation and overlap measures, and at times fuzzy correction for residue type exchangeability, all of which make difficult tracing the sources of their failure and success alike.

**Figure 3:**
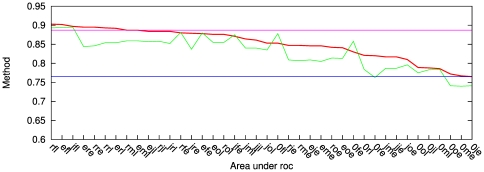
Combining various conservation and overlap scores into a single specificity scoring function for the LacI case. Method identifiers (see Methoda section and also Text S1): the first character: e: entropy, r: entropy modified by its expected value, j: Jensen-Shannon divergence from the stationary distribution, 0: no conservation score used. The second character: o: overlap of normalized distributions, f: squared difference, r: o modified by the expected value, m: pairwise mutual information. The third character: e: Euclidean distance, l: linear. Red: determinant model, green: discriminant. Pink: GroupSim, blue: mutual information. GroupSim uses conservation of neighboring residues as additional criterion. y-axis: area under the ROC curve for each method.

In Fig. 2 we show one particular choice of conservation and overlap methods discussed in the Methods section. However, other choices are possible, and indeed perform on the level within the noise bracket of the data. This is illustrated in Fig. 3, for the LacI test case. The remaining cases are relegated to supporting material. In the figure, all possible scores that can be obtained by combining the scoring and residue conservation - from literature, as well as proposed here - are listed on the x-axis in the order of decreasing area under the ROC curve. One striking feature, in this as well as in other test cases, is that with very few exceptions, for a given choice of scoring methods, the determinant model (red in Fig. 3) works better than discriminant (green).

### Protein-protein interaction

**Figure 4:**
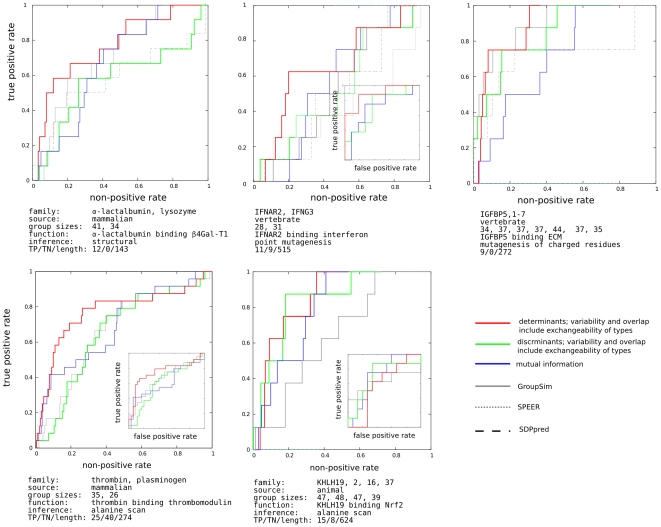
The same as Fig. 1, for protein-protein interaction cases.

Perhaps more interesting cases, where the difference between the determinant and discriminant behavior figures even more prominently, are the cases of specific interactions with proteins and other large polymers. The main descriptors for each test case - acquisition of interacting interface in 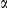-lactalbumin [56], he specificity of interferon-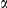 receptor for its favorite type of interferon, IGFBP5 specific binding to extracellular matrix [57], thrombin interface for thrombomodulin [58], and Kelch for Nrf2 [59] - are listed in panel captions in Fig. 4. The sequences used in the alignments, as well as the set of functional residues (as well as negative controls, when available) can be found in Materials S1. Mutual information systematically underperforms here, as do other methods that in one way or another incorporate the expectation of “constant-but-different” into their scoring function. Though a larger set of experimentally verified cases, at present difficult to build systematically, is certainly needed, the value of determinant approach is clearly illustrated.

## Discussion

In this work we have argued that a heuristic method to detect specificity in a set of paralogous proteins can be broken down to several independent components: (i) conservation (or variability) scoring function, (ii) overlap scoring function, (iii) the rule to add them together in a combined score, and, last but not least, (iv) the underlying model of evolution, specifying which groups are expected to be conserved, and which groups are expected (not) to overlap in the amino acid type choice. This disassembly of a heuristic scoring function enables tracking down the information contributing to the score, and discussing the merits of particular choice of its individual components. Some attention should be devoted to the model of evolution built therein - the siren call of symmetry across functionally divergent branches is a trap we easily fall into. To the contrary, it is easily demonstrable on the examples provided here (Fig. 3 and Text S1) that, with everything else kept the same, a method awarding determinant behavior may fare better than the one looking for discriminants. Stated plainly, positions of functional importance in one group need not be conserved in the groups of its paralogues.

Somewhat more puzzlingly, the linear combination of the scores has a tendency to perform better than the Euclidean one (Fig. 3 and Text S1), perhaps stemming simply from the even distribution of scores in the (conservation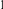,conservation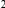, overlap) space.

Also, one of the outcomes of our investigation is the conclusion that, as intriguing as the assumption might seem, non-conserved, non-overlapping positions do not typically fall into the set of residues determining the functional divergence, and the scores not imposing the conservation as a requirement do not seem to represent a good strategy (the the scores withe systematically the lowest area under the ROC in Fig. 3 and Text S1.

We have also suggested a framework in which the evolution of each position on a peptide is modeled as an evolution of the distribution of amino acid types, and the strength of the evolutionary constraints is gauged by the difference of this distribution from the distribution the position would have, were it evolving free of constraints. In particular, this enabled us to modify the measure of overlap (which was somewhat elusive according to previous reports [30]) to accommodate our intuitive expectations on the exchangeability of amino acid types.

In our experiments with the scoring functions, we have demonstrated that the scoring functions that involve *some* degree of exchangeability of amino acid types fare better that the ones that include none (witness the behavior of “eo” function, standing for “plain entropy and overlap,” in Fig. 3 and Text S1). However, the available amount of experimental data does not presently allow us to prove that one way of treating conservation and overlap or including the exchangeability of amino acid types systematically outperforms the rest. Their different ranking in different examples indicates they are all within the noise bracket imposed by the underlying experiment, by the estimate of the average evolutionary behavior (Eq.2), and by the assumption of independent evolution of each site. We merely note that the description we offered in Eqs. 7 and 14 performs stably, and matches our intuitive expectations well.

Finally, one may ask, why bother with a heuristic approach which dispenses with the evolutionary tree, if ways for detailed description, including branching events, exist. The answer lies in its robustness, which allows one to deduce the gross features of evolutionary behavior that should be reproduced and bettered in development of a chronological model of evolution of a protein family. At the same time, the very lack of detailed features, in particular, of the order of the branching events leading to the observed set of sequences - which, if difficult to establish can be a source of noise itself - makes the approach applicable to a wide range of protein families, making them a useful cog in analysis pipelines.

The code used in the analysis is available from http://epsf.bmad.bii.a-star.edu.sg.

## Supporting Information

Text S1 **A pdf document, supp_info_text.pdf, containing additional figures and test set information.**
(PDF)

Materials S1 **A RAR archived directory, supp_info_materials.rar, containing the alignments used as test cases, and the positions therein of experimentally inferred specific residues.**
(RAR)
