# Supplementary material for: Determinants, Discriminants, Conserved Residues - A Heuristic Approach to Detection of Functional Divergence in Protein Families
Source: PLoS One. 2011 Sep 12;6(9):e24382. doi: 10.1371/journal.pone.0024382 (PMC3171465; doi:10.1371/journal.pone.0024382)
Supplement: Text S1 — A pdf document, supp_info_text.pdf, containing additional figures and test set information. (PDF) [file pone.0024382.s001.pdf]

SUPPLEMENT: DETERMINANTS, DISCRIMINANTS, CONSERVED RESIDUES - A HEURISTIC APPROACH TO DETECTION OF FUNCTIONAL DIVERGENCE IN PROTEIN FAMILIES

KAVITHA BHARATHAM, ZONG HONG ZHANG AND IVANA MIHALEK

Performance of various combinations of measures of conservation and overlap

Figs. 1,2, 3, 4, show areas under the ROC curve for various combinations of scoring and conservation scores discussed in main text. There, we use the following labeling scheme (on the x-axis) for the scoring functions, or, rather, their components:

- first character: conservation scoring function
  - **e**: Shannon entropy, Eq. 4 in the main text
  - **r**: Shannon entropy modified to include conservation, Eq. 7 in the main text
  - **j**: Jansen-Shannon divergence from stationary distribution, Eq. 3 in the main text
  - **0**: none
- second character: overlap scoring function
  - **o**: dot product of square normalized distributions, Eq. 8 in the main text
  - **r**: dot product of square normalized distributions, modified to include conservation Eq. 14 in the main text
  - **f**: sum of squared differences, Eq. 11 in the main text
  - **j**: Jansen-Shannon divergence between two groups, Eq. 12 in the main text
  - **m**: dot product of square normalized distributions, modified to include conservation Eq. 13 in the main text, with the index g running over two groups only
- third character: addition mode
  - **e**: Euclidean; Eq. 19 for discriminant model, and Eq. 20 for determinant
  - **l**: linear; Eq. 21 for discriminant model, and Eq. 22 for determinant

α-lactalbumin test case

This system provides an additional test of the ideas from the main text, but does not enjoy the strong experimental (mutational) backup as the other two cases. Instead we use an indirect but plausible structural argument, described below.

Lysozyme (c-type) and a-lactalbumin (LA) are structurally homologous proteins with distinct functions. Lysozyme is a lytic enzyme which degrades peptidoglycan, a constituent of bacterial cell walls [1], while LA interacts with galactosyltransferase to form lactose synthase complex, thereby altering the sugar acceptor specificity of galactosyltransferase to synthesize lactose in the mammary gland [2]. Their amino acid sequences [3] are highly similar, as is the intron-exon structure of their genes [4]. Therefore it is believed that lactalbumin arose from lysozyme by duplication of the gene. Subsequently, it acquired a new function, eventually loosing the old one. [5, 6].

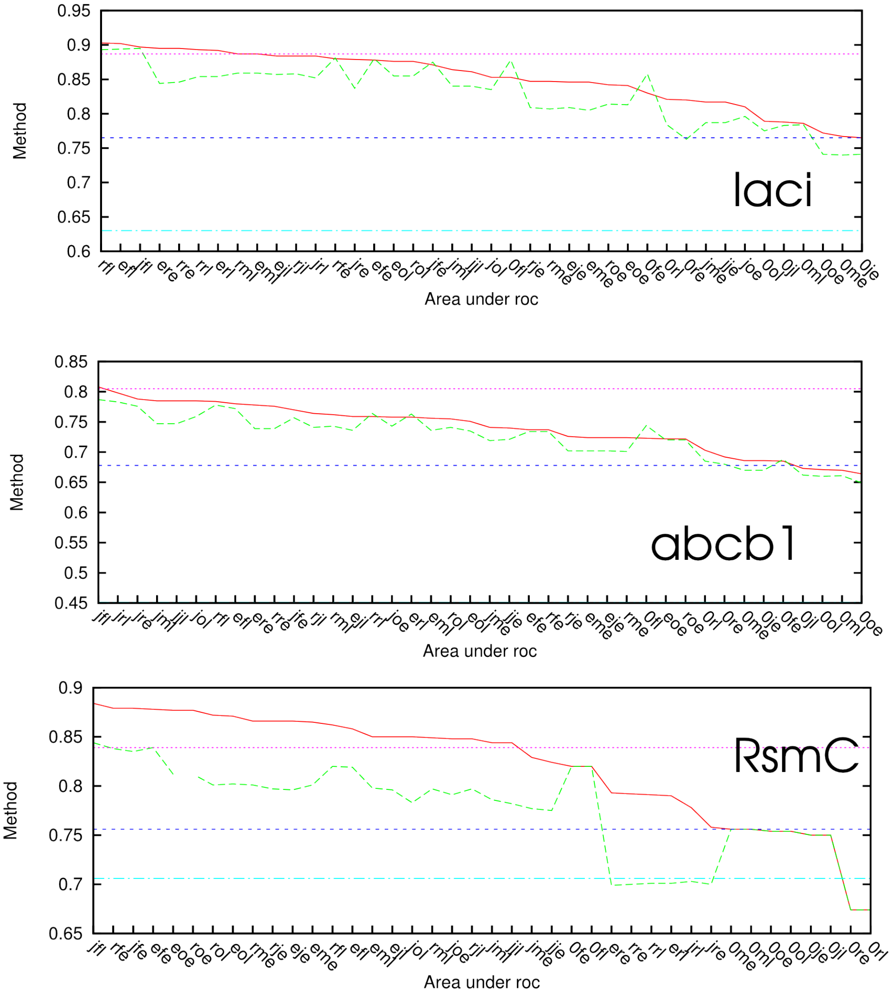

Figure 1. Areas under the ROC curve for small ligand test cases, for various ways of building the specialization measure from its main ingredients: conservation, overlap, distance function, and the evolutionary model. See the text of this supplement for the explanation of shorthands. Red: the models favoring determinants. Green: the models favoring discriminant positions. Blue mutual information. Pink: GroupSim. Cyan: SPEER. With very few exceptions, everything else being the same, determinant model outperforms the discriminant.

Catalytic domain of bovine beta 1, 4-galactosyltransferase was crystallized in complex with alpha-lactalbumin by Ramakrishnan *et al.* [7], and deposited in PDB under id 2fyd. We used the geometric information therein, to list the residues on lactalbumin such that the atoms of their sidechains can be found within $4\mathring{A}$ from the galactosyltransferase. Since the capability of forming this interface is irrelevant for lysozyme, we used the as the set of residues giving functional specificity to lactalbumin.

For both genes - lactalbumin and lysozyme - we used all orthologues available from Ensembl(http://www.ensembl.org/index.html), established to be so by their one-to-one correspondence to the human gene. The nature of lactalbumin dictated that the set of sequences be restricted to mammals.

LacI test case

The residues considered to be specificity determining for LacI (group XI in [8]) were the following, according to the numbering in E. coli LacI: L73, A75, I79, N125, P127, D149, S191, S193, W220, N246, Q248, Y273, T276, F293. Two more residues were quoted by Suckow *et al.* as affecting the effector binding: R197 and D274. The two are completely conserved across all three groups of proteins, and would thus, by most authors, not be counted as members of the specificity determining group of residues.

The sequences for all 3 groups of orthologs (GalR, PurR, LacI) were retrieved by gene name search from Uniprot ([9]; http://www.uniprot.org/). Each group was aligned separately using Muscle [10], and sequences that were not within 20% of identity from all other sequences were removed (making sure E.coli versions of the protein are in the cluster), as were the fragments shorter than 2/3 of the length of the E.coli. Individual family alignments were aligned to each other using the profile mode of alignment in Mafft.

IFNAR2 test case

The data is from Piehler adn Schreiber [11].

- M48(73) hotspot - 15kJ/mol of binding energy
- T46(71)A, I47(72)A decreased the affinity for IFN-α by more then tenfold
- E79A, I105(130)A decreased the affinity for IFN-α by 4 to 5-fold
- S49(74)A, K50(75)A, W74F, H78A, W102(127)A decreased the affinity for IFN-α by 2 to 3-fold
- E52(77)A, K55(80)A and S76A decreased the affinity less then 2fold, suggesting periphery of the binding site, at best
- D33, E55, D78, D97, T102, E111, N125, E159, D165, H214, E217 did not change the affinity to any measurable extent

We chose to use as positives only the positions causing the increase in affinity of more than twofold. W102A looks a bit suspicious, because it seems to fall in a highly variable region (see the alignment region in Fig. 5). The last group of residues, causing no measurable change in affinity (upon mutation), was used as the set of "negatives."

For both genes, we used all orthologues available from Ensembl (http://www.ensembl.org/index.html), established to be so by their one-to-one correspondence to the human gene. These two proteins exhibit lot of variability, even within vertebrates. To ensure reliable alignment, we limited the species selection, in both groups, to mammals. This alignment can be downloaded from http://epsf.bmad.bii.a-star.edu.sg/.

IGFB5 test case

True positives: positions changing th relative affinity of IGFBP5 for ECM by more than 40%. (Clemmons *et al.* [12], Table 1

Kelch test case

True positives: positions significantly impairing Nrf2-dependent repression. (Lo *et al.* [13], Table1.)

Thrombin test case

True positives: affinity for thrombomodulin due to mutation, expressed as $\log K_{d,mut}/K_{d,wt}$ or the change in the specificity constant for recognizing protein C due to mutation, expressed as $\log s_{mut}/s_w$, greater than 0.5. Xu *et al.* [14], Fig. 1). The rest of residues in that figure were taken to be true negatives.

ABCB1 test case

True positives: upon mutation cause more than twofold decrease in resistance to to one or more drugs. (Hanna *et al.*, [15], Fig. 3 and the rated discussion in the text.)

RsmC test case

True positives: upon mutation decrease the MTase activity by more than 50%. (Sunita *et al.* [16], Fig.4)

Miscelaneous

- All measures of conservation used in the text received the same treatment: they were scaled to $[0, 1]$ range, so that the smallest value seen across all positions within paralogous group equals 0, and the largest one equals 1.
- All measures of overlap used in the text received the same treatment: they were scaled to $[0, 1]$ range, so that the smallest value seen across all positions and between any paralogous group equals 0, and the largest one equals 1.
- Treatment of gaps in the alignment. Positions with more than 30% of gaps in any group were ignored. None of such positions appeared in our sets of specific residues, though in principle, inserts are good candidates for specific positions. We believe it is beyond the scope of discussion presented here. Otherwise, gaps were ignored, and the frequency of amino acid types estimated based on the non-gapped positions.
- Treatment of missing residues. Missing or unknown residues, indicated in the source by "X" (rather than as a gap) were treated as being equal to the type seen in the most similar sequence from the same group, provided that the sequence is at least 40% identical and has at least 40% of the length of the sequence with the unknowns. If no such neighbor could be found, the position was ignored, and the frequency of amino acid types estimated based on the non-gapped positions.
- all alignments used in this work were created using Mafft [17], and restricted to the positions in the representative sequence, for easier comparison across different methods (and programs that implement them)

References

[1] Fleming A (1922) On a remarkable bacteriolytic element found in tissues and secretions. Proceedings of the Royal Society of London Series B, Containing Papers of a Biological Character 93: 306–317.

[2] Hill R, Brew K (1975) Lactose synthetase. Advances in enzymology and related areas of molecular biology 43: 411.

[3] Brew K, Castellino F, Vanaman T, Hill R (1970) The complete amino acid sequence of bovine α-lactalbumin. Journal of Biological Chemistry 245: 4570.

[4] Qasba P, Safaya S (1984) Similarity of the nucleotide sequences of rat α-lactalbumin and chicken lysozyme genes. Nature 308.

[5] Nitta K, Sugai S (1989) The evolution of lysozyme and α-lactalbumin. European Journal of Biochemistry 182: 111–118.

[6] Prager E, Wilson A (1988) Ancient origin of lactalbumin from lysozyme: analysis of DNA and amino acid sequences. Journal of Molecular Evolution 27: 326–335.

[7] Ramakrishnan B, Ramasamy V, Qasba P (2006) Structural Snapshots of [beta]-1, 4-Galactosyltransferase-I Along the Kinetic Pathway. Journal of molecular biology 357: 1619–1633.

[8] Suckow J, Markiewicz P, Kleina L, Miller J, Kisters-Woike B, et al. (1996) Genetic studies of the lac repressor XV: 4000 single amino acid substitutions and analysis of the resulting phenotypes on the basis of the protein structure. Journal of molecular biology 261: 509–523.

[9] Boeckmann B, Bairoch A, Apweiler R, Blatter MC, Estreicher A, et al. (2005) The universal protein resource (uniprot) 33: D154-D159.

[10] Edgar R (2004) Muscle: multiple sequence alignment with high accuracy and high throughput 32: 1792-97.

[11] Piehler J, Schreiber G (1999) Mutational and structural analysis of the binding interface between type I interferons and their receptor ifnar2. Journal of molecular biology 294: 223–237.

[12] Clemmons D (2001) Use of mutagenesis to probe igf-binding protein structure/function relationships. Endocrine reviews 22: 800.

[13] Lo S, Li X, Henzl M, Beamer L, Hannink M (2006) Structure of the keap1: Nrf2 interface provides mechanistic insight into nrf2 signaling. The EMBO Journal 25: 3605–3617.

[14] Xu H, Bush L, Pineda A, Caccia S, Di Cera E (2005) Thrombomodulin changes the molecular surface of interaction and the rate of complex formation between thrombin and protein c. Journal of Biological Chemistry 280: 7956.

[15] Hanna M, Brault M, Kwan T, Kast C, Gros P (1996) Mutagenesis of transmembrane domain 11 of p-glycoprotein by alanine scanning. Biochemistry 35: 3625–3635.

[16] Sunita S, Purta E, Durawa M, Tkaczuk K, Swaathi J, et al. (2007) Functional specialization of domains tandemly duplicated within 16s rrna methyltransferase rsmc. Nucleic acids research 35: 4264.

[17] Katoh K, Kuma K, Toh H, Miyata T (2005) Mafft version 5: improvement in accuracy of multiple sequence alignment. Nucleic acids research 33: 511.

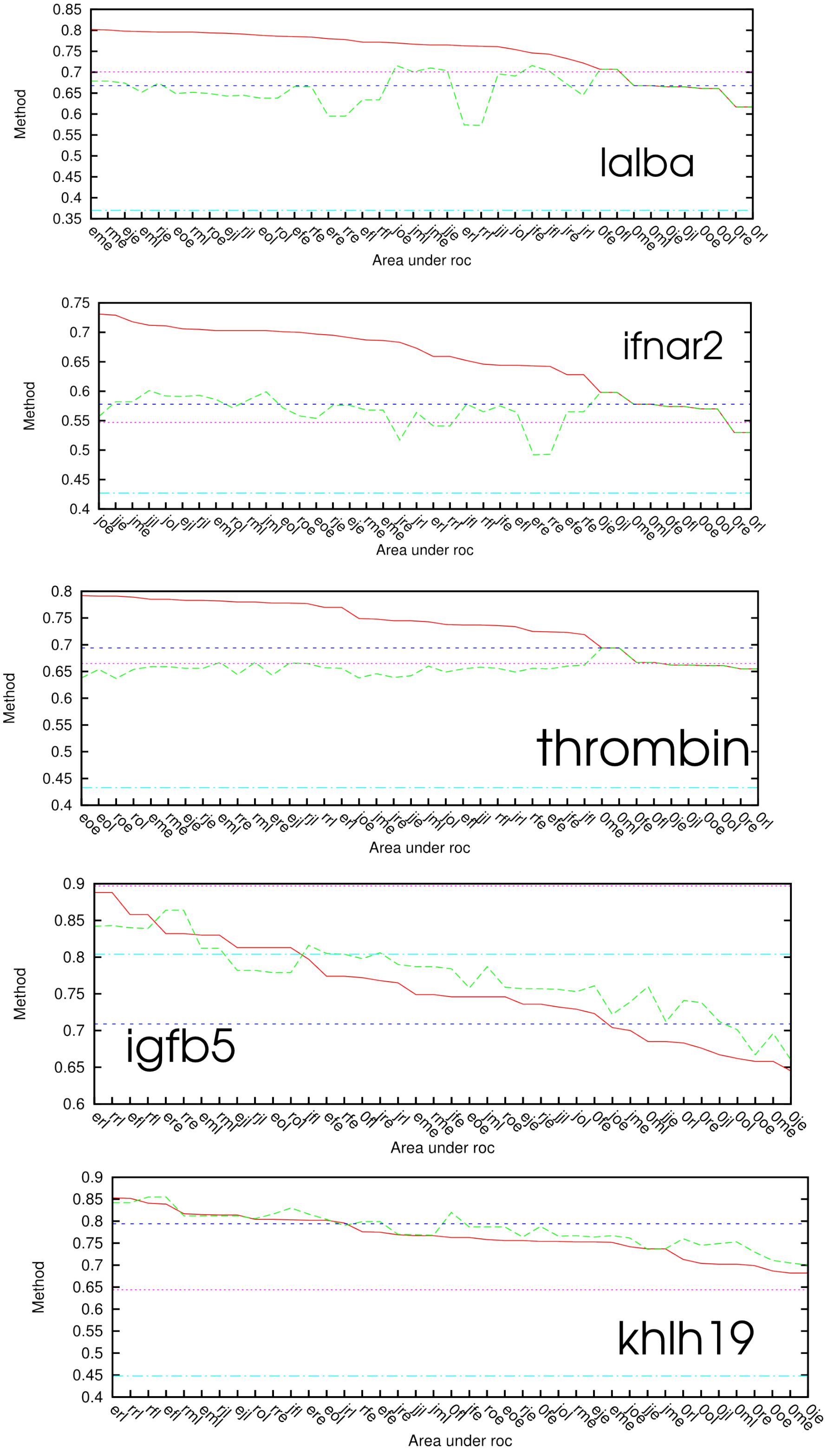

Figure 2. The same as 1 for the test cases involving protein-protein interaction.

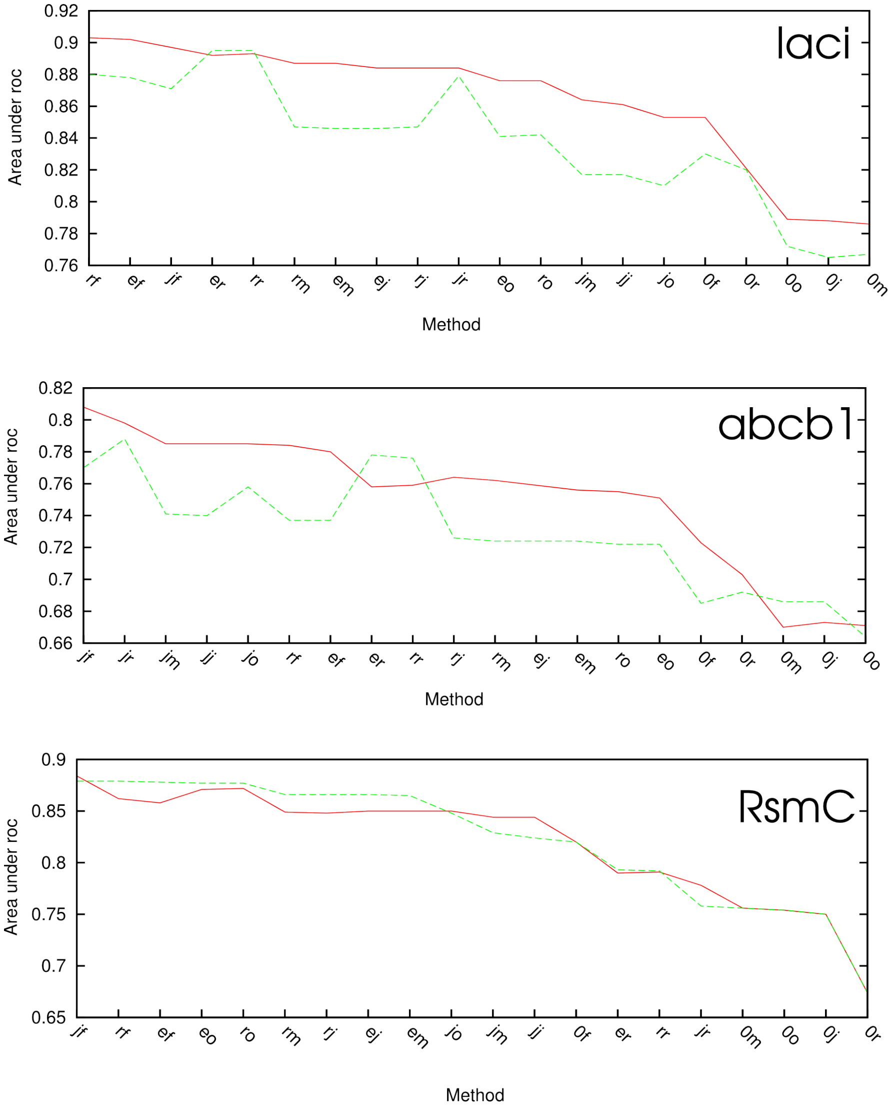

Figure 3. Small ligand test cases. Linear (red) vs. euclidean (green) distance, for the same pair of of conservation and overlap measures given on the x-axis. With a few exceptions, when the difference is minor, the linear combination seems to be a better choice.

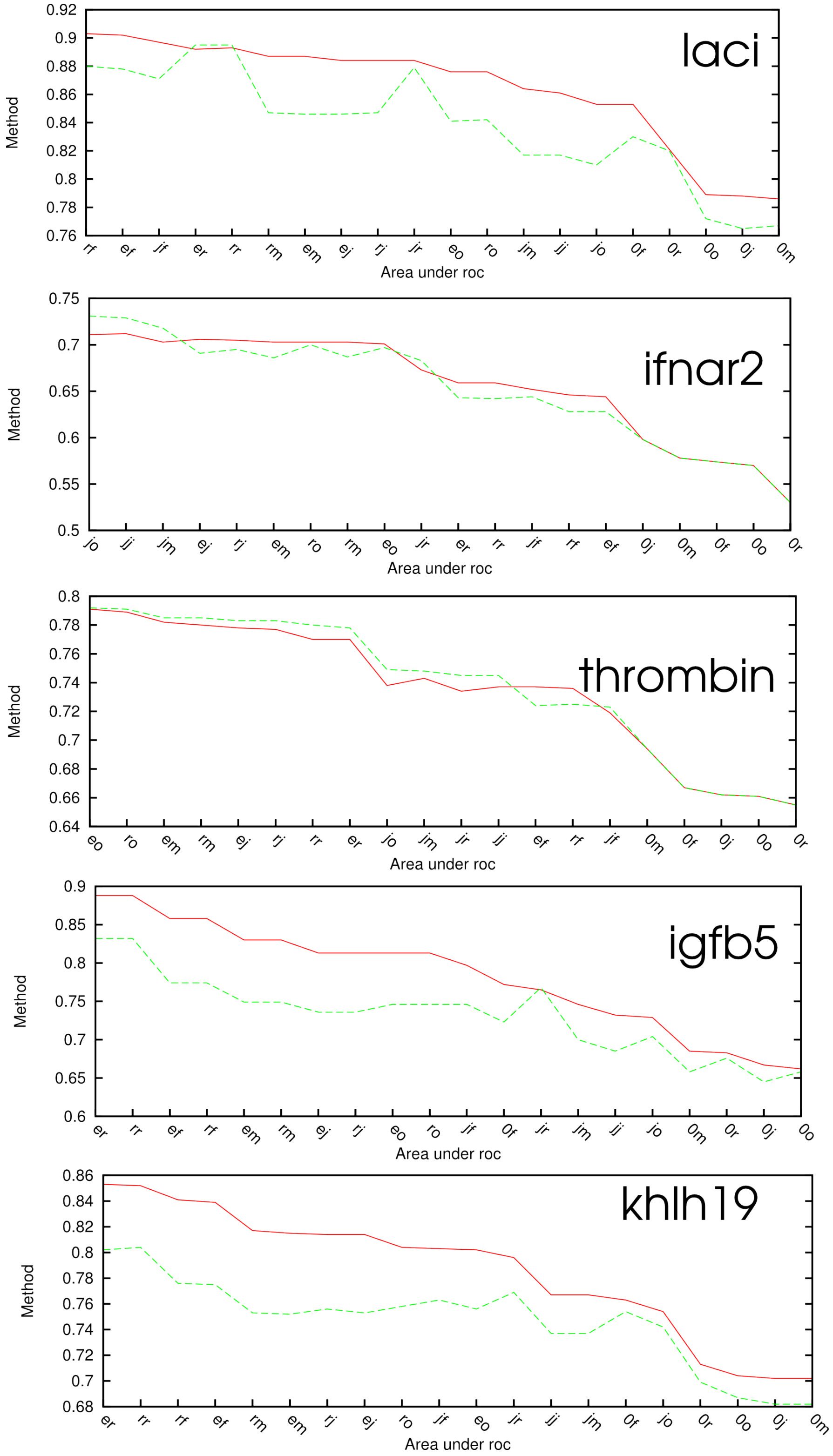

Figure 4. The same as Fig. 3 for protein-protein interaction test cases.

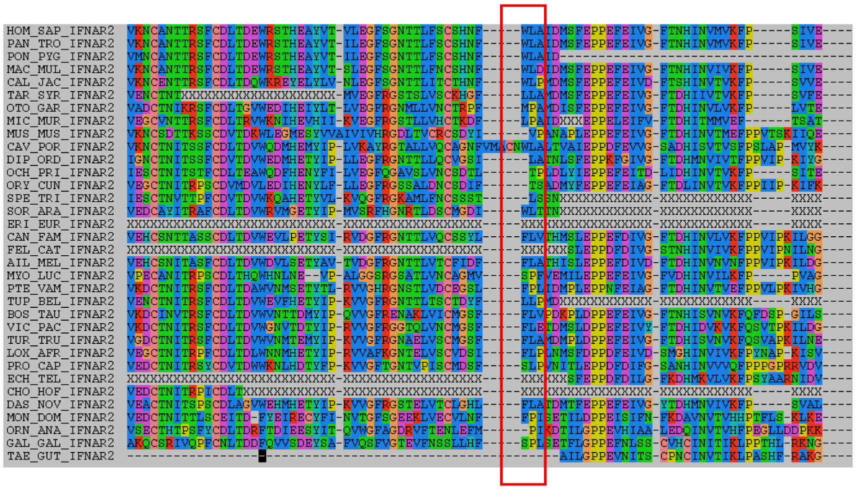

Figure 5. The region of the IFNAR2/IFNGR1 alignment around W102. Notable is the high variability therein.
